# Early-Onset Colorectal and Gastric Adenocarcinomas in a Patient With a Heterozygous BLM p.E1317K Variant of Uncertain Significance

**DOI:** 10.7759/cureus.111459

**Published:** 2026-06-24

**Authors:** Arti A Patel, Victor M Samperio, Henry T Tsai

**Affiliations:** 1 Department of Internal Medicine, Eisenhower Medical Center, Rancho Mirage, USA; 2 Department of Medicine, Eisenhower Medical Center, Rancho Mirage, USA; 3 Department of Hematology/Oncology, Eisenhower Medical Center, Rancho Mirage, USA

**Keywords:** blm gene, colorectal cancer, gastric cancer (gc), h pylori, microsatellite stability

## Abstract

Early-onset gastrointestinal malignancies may raise concerns for hereditary cancer susceptibility, although interpretation of rare germline variants identified through multigene testing remains challenging. We describe the case of a male patient diagnosed with stage IIIB mismatch repair-proficient sigmoid adenocarcinoma at age 29 who subsequently developed a poorly differentiated gastric adenocarcinoma five years later. Germline testing identified a heterozygous BLM p.E1317K variant of uncertain significance (VUS) and no other pathogenic germline alterations. Both tumors demonstrated microsatellite-stable, mismatch repair-proficient disease with low tumor mutational burden, and the patient’s family history was notable for gastric cancer in his father, collectively raising concern for an underlying cancer predisposition. Although Bloom syndrome is associated with biallelic pathogenic BLM variants, the oncologic significance of heterozygous BLM alterations remains incompletely defined. This report highlights the interpretive challenges surrounding rare germline variants in patients with multiple early-onset gastrointestinal malignancies and underscores the need for further investigation into the potential role of selected heterozygous BLM variants in cancer susceptibility.

## Introduction

Colorectal cancer (CRC) and gastric cancer are among the most commonly diagnosed malignancies worldwide, ranking third and fifth, respectively [1,2]. While the overall incidence of CRC has declined among older populations following the widespread implementation of screening programs, the incidence of early-onset CRC continues to rise [3]. Although most gastrointestinal malignancies occur sporadically, the occurrence of multiple primary tumors in a young patient is uncommon and may suggest an underlying hereditary cancer predisposition syndrome or other biological contributors to carcinogenesis [4]. Concurrently, with the growing incorporation of multigene germline testing into routine oncologic practice, clinicians are increasingly encountering rare germline variants whose clinical significance remains uncertain because of limited functional characterization and the paucity of reported cases needed to establish definitive genotype-phenotype associations [5,6].

BLM encodes a RecQ helicase involved in DNA replication, homologous recombination repair, and maintenance of genomic stability [7]. Biallelic pathogenic BLM variants cause Bloom syndrome, a rare autosomal recessive chromosomal instability disorder characterized by photosensitivity, immune dysfunction, growth impairment, and a markedly increased risk of early-onset malignancy, including gastrointestinal cancers such as CRC [8]. Gastrointestinal cancers, particularly CRC, have been described in Bloom syndrome [8]. In contrast, the oncologic significance of heterozygous BLM variants remains incompletely defined. Several studies have suggested a possible association between selected heterozygous BLM alterations and CRC susceptibility, although available evidence remains limited and inconsistent [9-11].

We report the case of a young male with mismatch repair-proficient sigmoid adenocarcinoma diagnosed at age 29 who subsequently developed primary gastric adenocarcinoma five years later and was found to carry a heterozygous BLM p.E1317K variant of uncertain significance (VUS) in the setting of a paternal history of gastric cancer. This report highlights the interpretive challenges surrounding rare germline variants identified during hereditary cancer evaluation and underscores the importance of integrating molecular findings with clinical phenotype, family history, and tumor biology.

## Case presentation

A 29-year-old male with a history of intermittent hematochezia and prior benign colon polyps presented with worsening bright red blood per rectum, symptomatic anemia (Table 1), abdominal cramping, and intermittent lightheadedness. He reported experiencing intermittent hematochezia for approximately two years before presentation. His family history was notable for gastric cancer in his father. He denied significant alcohol consumption, tobacco use, recreational drug use, or relevant occupational exposures.

**Table 1:** Initial laboratory values CEA: carcinoembryonic antigen; MCV: mean corpuscular volume

Laboratory test	Result	Normal range
Hemoglobin	7.5 g/dL	14.0 - 18.0 g/dL
Hematocrit	28.6%	42.0 - 54.0%
MCV	58.4 fL	80.0 - 99.0 fL
CEA	1.1 ng/mL	0.0 - 4.7 ng/mL

Colonoscopy demonstrated a circumferential, near-obstructing mass preventing visualization of the distal colon. Biopsy showed moderately differentiated adenocarcinoma with inflammatory polyps suggestive of ulcerative colitis. Carcinoembryonic antigen (CEA) was normal at 1.1 ng/mL (Table 1). CT demonstrated segmental sigmoid wall thickening with pericolonic inflammatory and nodular changes concerning for malignancy, without evidence of metastatic disease. The following month, the patient underwent laparoscopic hand-assisted left colectomy with coloproctostomy and omental flap placement. Final pathology demonstrated pT4a pN1a M0 stage IIIB well-differentiated sigmoid adenocarcinoma with visceral peritoneal invasion and an involved radial/mesenteric margin. Lymphovascular and perineural invasion were absent.

Mismatch repair immunohistochemistry demonstrated intact MLH1, MSH2, MSH6, and PMS2 expression. Tumor proportion score for programmed death-ligand 1 (PD-L1) was 0%. Next-generation sequencing (NGS) demonstrated KRAS G12D, TP53 R248W, BRCA2 S1650fs*20, and CTNNB1 splice-site alterations. The tumor was microsatellite stable with a low tumor mutational burden of three mutations/megabase. NRAS was wild type. Germline multigene testing identified a heterozygous BLM p.E1317K VUS, without additional pathogenic germline alterations. The BRCA2 alteration detected in the tumor was not identified on germline testing, suggesting a somatic origin.

The patient completed adjuvant modified FOLFOX-6. Initial surveillance imaging demonstrated no evidence of recurrence. During follow-up, however, he developed recurrent iron-deficiency anemia (Table 2) with intermittent melena and hematochezia. Circulating tumor DNA (ctDNA) subsequently became positive despite negative imaging and normal CEA levels (Table 2), raising concerns for occult disease.

**Table 2:** Follow-up laboratory values showing iron deficiency anemia, normal CEA, and positive ctDNA CEA: carcinoembryonic antigen; ctDNA: circulating tumor DNA; MCV: mean corpuscular volume

Laboratory test	Result	Normal range
Hemoglobin	9.2 g/dL	14.0 - 18.0 g/dL
Hematocrit	34.7%	42.0 - 54.0%
MCV	63 fL	80.0 - 99.0 fL
Iron-binding capacity	416 ug/dL	250 - 450 ug/dL
Unsaturated iron-binding capacity	400 ug/dL	11 - 343 ug/dL
Iron	16 ug/dL	38 - 169 ug/dL
Iron saturation	4%	15 - 55%
Ferritin	4 ng/dL	30 - 400 ng/dL
CEA	<0.03 ng/mL	0.0 - 4.7 ng/mL
ctDNA	Positive	Undetectable

Further gastrointestinal evaluation demonstrated persistent inflammatory and polypoid pathology. Colonoscopy showed multiple inflamed colonic polyps, a sessile serrated adenoma, and focal active colitis. Upper endoscopy later revealed multiple gastric polyps, chronic gastritis, *Helicobacter pylori (H. pylori)* infection, and adenomatous gastric mucosa with high-grade dysplasia involving the incisura. *H. pylori* eradication therapy was completed, with subsequent testing confirming eradication. Despite intravenous iron supplementation, recurrent iron-deficiency anemia and gastrointestinal bleeding persisted.

**Figure 1:**
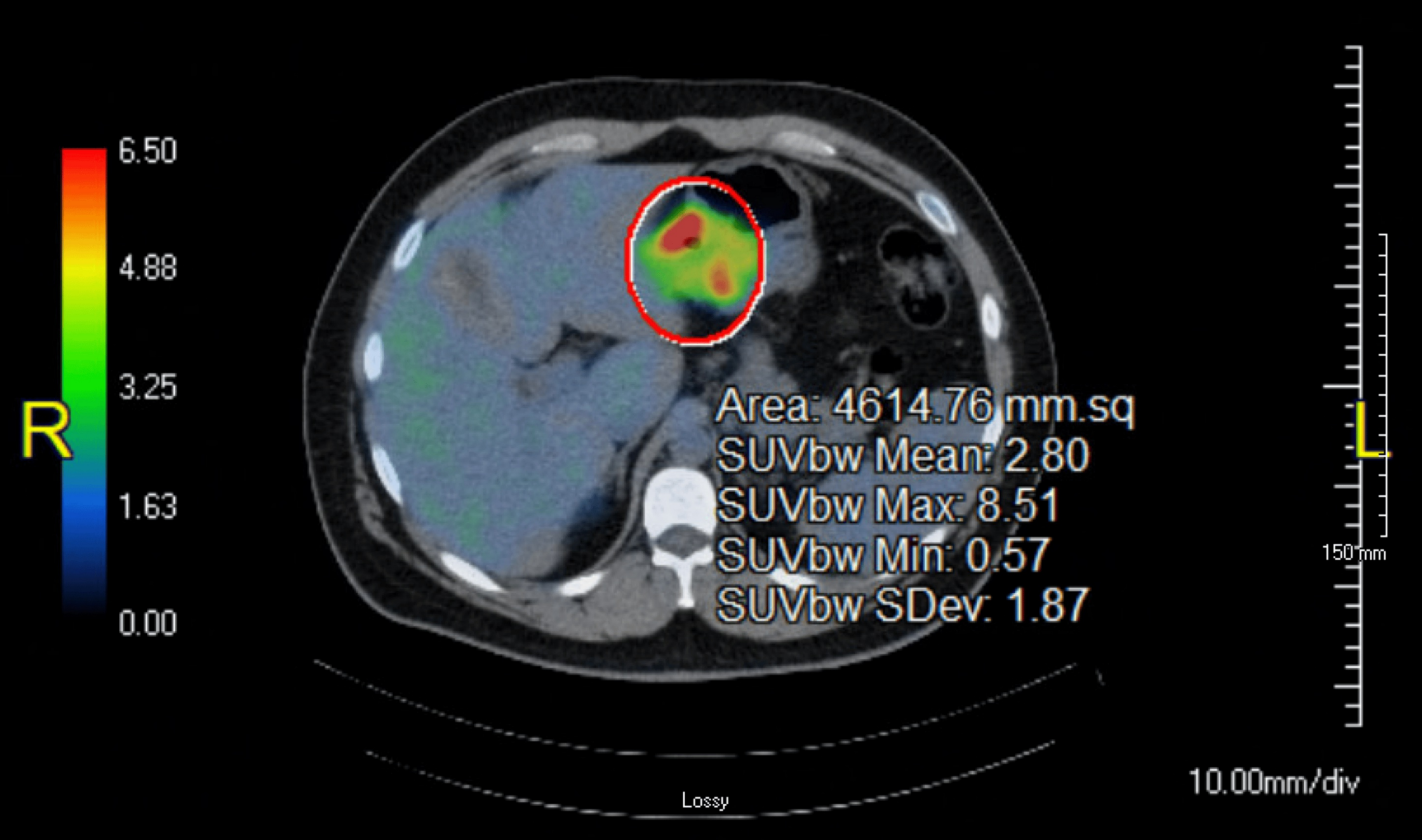
PET scan showing an annular mass in the gastric antrum with an area of approximately 75 mm, with maximum SUV of 8.5 PET: positron emission tomography; SUV: standardized uptake value

**Figure 2:**
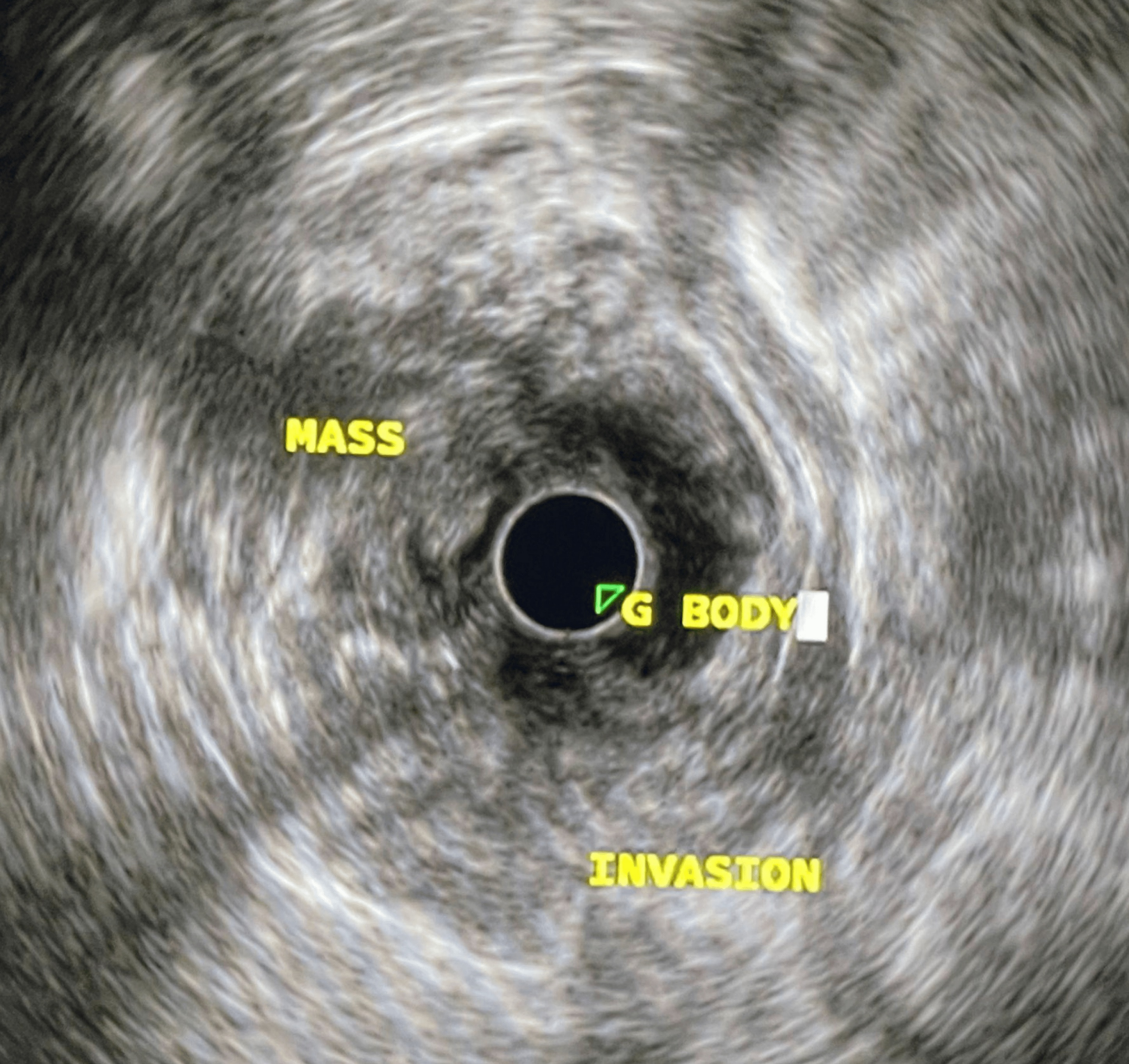
Endoscopic ultrasound showing an invasive gastric mass

Approximately five years after the initial diagnosis of CRC, positron emission tomography demonstrated a large fluorodeoxyglucose-avid mass occupying most of the gastric antrum (Figure 1). Endoscopic ultrasound revealed a large exophytic multilobulated mass extending from the pyloric channel to the gastric midbody measuring approximately 6-7 cm, with an additional separate prepyloric lesion measuring approximately 2 cm and irregular mucosal abnormalities involving the lesser curvature (Figure 2). Biopsy confirmed poorly differentiated gastric adenocarcinoma arising in association with villotubular adenoma and high-grade dysplasia.

Tumor profiling of the gastric malignancy demonstrated HER2-negative disease, intact mismatch repair protein expression, microsatellite-stable status, and low tumor mutational burden of 4.2 mutations/megabase. PD-L1 tumor proportion score was less than 1%, with a combined positive score (CPS) of 1. Molecular analysis identified genomic alterations including KRAS p.G12D, CTNNB1 p.N387K, SMAD4 p.R445, and FAS p.E194fs, along with copy number losses involving AMER1, CDKN2A, CDKN2B, and MTAP.

The patient subsequently received perioperative FLOT chemotherapy consisting of docetaxel, oxaliplatin, fluorouracil, and leucovorin. Following six cycles, he underwent total gastrectomy with Roux-en-Y esophagojejunostomy and feeding jejunostomy. Surgical pathology demonstrated a focal intramucosal adenocarcinoma arising in a background of adenoma with high-grade dysplasia, consistent with a marked treatment response, with 27 negative lymph nodes and a final staging of pTis N0. Postoperative circulating tumor DNA testing was negative, and surveillance imaging showed no definitive evidence of active disease. The patient continues under active surveillance and monitoring.

This case describes a young patient with metachronous colorectal and gastric adenocarcinomas, recurrent premalignant gastrointestinal lesions, persistent iron-deficiency anemia, a family history of gastric cancer, and a heterozygous BLM VUS despite otherwise unrevealing germline testing.

## Discussion

**Figure 3:**
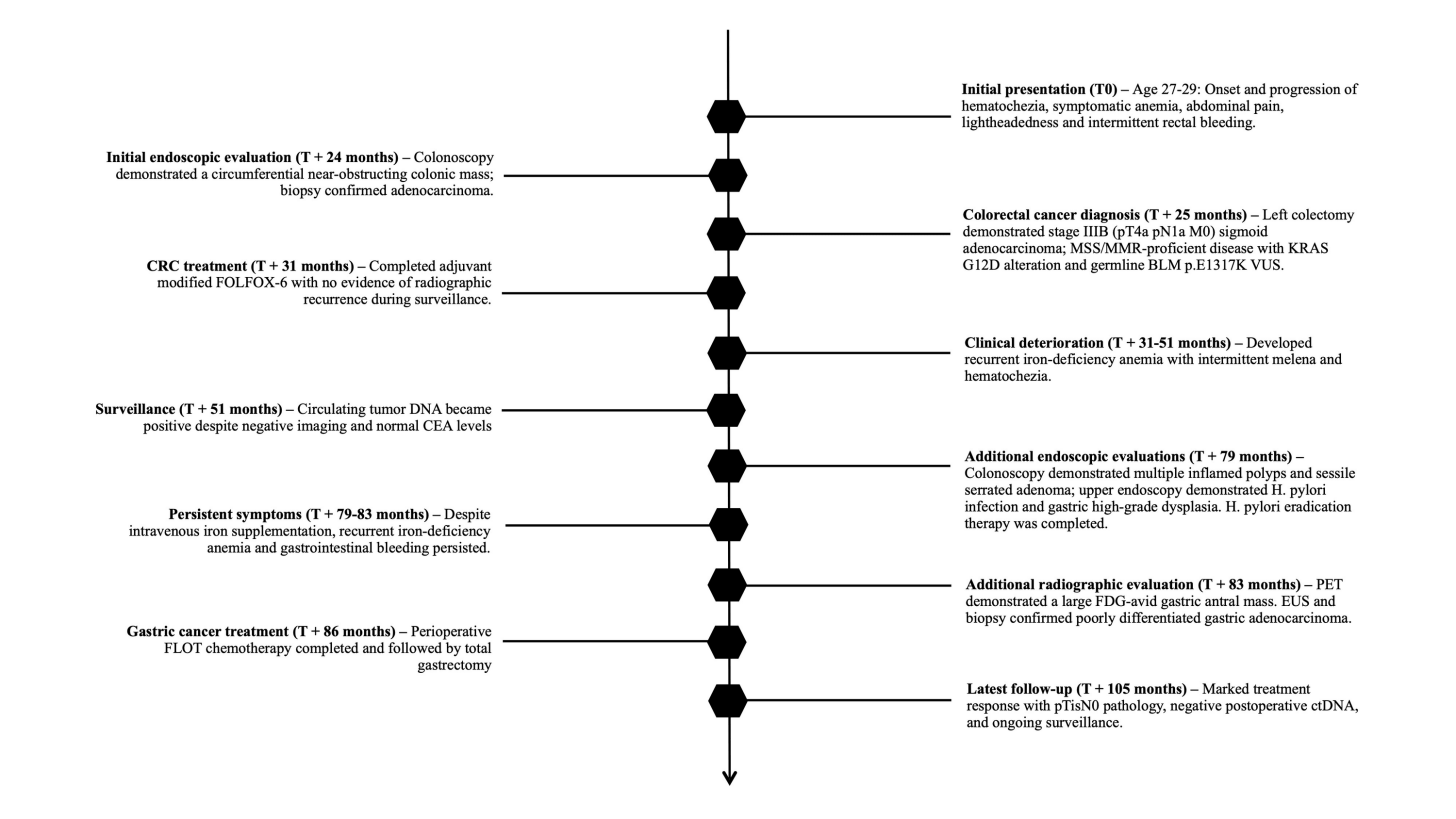
Clinical timeline of key diagnostic and therapeutic events illustrating progression from early-onset colorectal adenocarcinoma to subsequent gastric adenocarcinoma T indicates time relative to initial presentation (T0), where T0 denotes the time of initial presentation BLM: Bloom syndrome protein gene; CEA: carcinoembryonic antigen; ctDNA: circulating tumor DNA; EUS: endoscopic ultrasound; FDG: fluorodeoxyglucose; KRAS: Kirsten rat sarcoma viral oncogene homolog; MMR: mismatch repair; MSS: microsatellite stable; PET: positron emission tomography; VUS: variant of uncertain significance

This case illustrates the diagnostic and interpretive complexity of multiple early-onset gastrointestinal malignancies occurring in the setting of rare germline variation. The patient developed stage IIIB sigmoid adenocarcinoma at age 29 and subsequently developed primary gastric adenocarcinoma approximately five years later (Figure 3). The combination of early-onset CRC, a second primary gastric cancer, paternal history of gastric cancer, and otherwise unrevealing germline testing raises concern for an underlying cancer predisposition. However, the heterozygous BLM p.E1317K variant identified in this patient remains a VUS and cannot be considered causative based on currently available evidence.

Although selected heterozygous BLM alterations have been associated with increased CRC susceptibility in some studies, particularly among patients with early-onset disease [9,10], current evidence remains insufficient to classify BLM as a definitive autosomal dominant hereditary CRC syndrome [11].

Several molecular features of this case are notable. Both tumors were mismatch repair-proficient, microsatellite stable, and associated with low tumor mutational burden, making a Lynch syndrome-associated phenotype less likely. Both malignancies also harbored KRAS G12D alterations; however, KRAS mutations commonly occur across gastrointestinal cancers and are unlikely to independently explain the broader phenotype of early-onset dual primary malignancies, recurrent premalignant gastrointestinal pathology, and paternal gastric cancer history. Additional tumor-specific alterations involving TP53, CTNNB1, SMAD4, and CDKN2A/B likely reflect independent tumor evolution rather than a unifying inherited mechanism.

The broader clinical phenotype may ultimately be more informative than the isolated variant finding itself. The patient developed recurrent inflammatory and premalignant gastrointestinal pathology, including sessile serrated lesions, gastric high-grade dysplasia, and subsequent gastric adenocarcinoma despite initially unrevealing surveillance studies. An underlying inflammatory bowel disease was considered; however, autoimmune serologies were negative, and histopathologic evaluation did not demonstrate features of chronic inflammatory bowel disease. *H. pylori* infection may also have contributed to gastric carcinogenesis and should not be disregarded, given its established role as a gastric cancer risk factor [12]. Furthermore, the specific BLM p.E1317K alteration identified in this patient was classified as a VUS in ClinVar at the time of review [13]. Therefore, despite the patient’s compelling phenotype, the identified variant should be interpreted cautiously as a possible susceptibility signal rather than evidence of a confirmed hereditary syndrome.

This case does not establish pathogenicity of BLM p.E1317K. No segregation analysis, functional assay, loss-of-heterozygosity assessment, or second-hit evaluation was available. The gastric tumor also did not demonstrate known biallelic BLM inactivation. Experimental models provide biologic plausibility for a role of altered BLM dosage in tumor susceptibility: heterozygous Blm mice demonstrated increased tumor formation, Bloom mouse models showed cancer predisposition associated with elevated mitotic recombination, and reduced BLM protein levels correlated with chromosomal instability and tumor predisposition [14-16]. However, these findings derive primarily from experimental systems and cannot be directly extrapolated to the clinical significance of the specific p.E1317K variant identified in this patient. These limitations preclude causal inference.

As multigene germline testing becomes increasingly integrated into routine oncologic practice, clinicians will continue to encounter patients with compelling cancer histories and rare VUS findings. This report highlights that patients with clinically suspicious phenotypes may still warrant individualized surveillance strategies and comprehensive genetic counseling despite nondiagnostic germline findings. Further studies incorporating functional characterization, segregation analyses, and tumor-based assessment are needed to clarify whether selected heterozygous BLM variants contribute meaningfully to gastrointestinal cancer susceptibility.

## Conclusions

This case report described a young male with early-onset mismatch repair-proficient sigmoid adenocarcinoma and subsequent microsatellite-stable gastric adenocarcinoma found to carry a heterozygous BLM p.E1317K VUS. Although the clinical presentation raised suspicion for an underlying predisposition to gastrointestinal malignancy, current evidence is insufficient to classify this variant as pathogenic or to support variant-directed screening recommendations. The findings should therefore be interpreted as hypothesis-generating. It underscores the importance of integrating germline findings within the broader clinical context and supports further investigation into the potential role of selected heterozygous BLM variants in early-onset gastrointestinal cancer susceptibility.
